# Determination of the Chemical Composition and Antimicrobial Activity of *Lavatera thuringiaca* L. Medicinal Herb Material Extracted under Subcritical Conditions by the Liquid Carbon Dioxide Method

**DOI:** 10.1155/2021/7541555

**Published:** 2021-07-21

**Authors:** Moldir A. Zhandabayeva, Kaldanay K. Kozhanova, Assyl K. Boshkayeva, Valeriy A. Kataev, Gulbaram O. Ustenova, Nadezhda G. Gemejiyeva, Zhanar A. Iskakbayeva

**Affiliations:** ^1^Department of Engineering Disciplines, Asfendiyarov Kazakh National Medical University, Almaty 050000, Kazakhstan; ^2^Department of Pharmaceutical and Toxicological Chemistry, Pharmacognosy and Botany, Asfendiyarov Kazakh National Medical University, Almaty 050000, Kazakhstan; ^3^Pharmaceutical Faculty, Bashkortostan State Medical University, Ufa 450000, Russia; ^4^Department of Pharmaceutical Technology, Asfendiyarov Kazakh National Medical University, Almaty 050000, Kazakhstan; ^5^Laboratory of Plant Resources, Institute of Botany and Phyto-Introductions, Almaty 050057, Kazakhstan; ^6^Laboratory of Microbiology, Scientific Center of Anti-Infectious Drugs, Almaty 050057, Kazakhstan

## Abstract

This article presents the composition of the components of *Lavatera thuringiaca* L. (*Malvaceae* Juss. family), which has a certain antibacterial effect. The plant collection was carried out in the Shamalgan gorge of Mountain Range of the Trans-Ili Alatau in the territory of the Karasay district of the Almaty region, in the flowering phase. A CO_2_ extract of the aboveground part of the medicinal plant *Lavatera thuringiaca* L. was obtained under subcritical conditions and, for the first time, studied for its component composition and antimicrobial activity. Determination of the chemical composition of the extract was carried out by gas chromatography/mass spectrometry (GC/MS). To identify the obtained mass spectra, we used the Wiley 7^th^ edition and the NIST'02 data library. To determine the antimicrobial and antifungal activity, standard test strains of microorganisms were used: *Staphylococcus aureus* ATCC 6538-P, *Escherichia coli* ATCC 8739, *Pseudomonas aeruginosa* ATCC 9027, *Candida albicans* ATCC 10231, *Streptococcus pneumonia* ATCC 660, *Klebsiella pneumoniae* ATCC 700603, *Staphylococcus haemolyticus*, and *Staphylococcus saprophyticus*. In the composition of thick CO_2_*Lavatera thuringiaca* L. extract, the content of 31 components was proven: spathulenol 6.97%, pulegone 5 08%, cis-*β*-farnesene 7.63%, verbenone 1.93%, *α*-bisabolol oxide B 9.65%, bisabolol oxide A 8.26%, *α*-bisabolol 1.36%, linolenic acid, ethyl ether 3.15%, phytol 2.49%, herniarin 5.61%, linolenic acid 9.38%, linoleic acid 6.95%, myristic acid 2.33%, and elaidic acid 2.57%. Antimicrobial activity studies have shown that the CO_2_ extract of *Lavatera thuringiaca* L. has a pronounced effect against clinically significant microorganisms: *Staphylococcus aureus*, *Escherichia coli*, *Pseudomonas aeruginosa*, *Candida albicans*, *Streptococcus pneumonia*, *Klebsiella pneumoniae*, *Staphylococcus haemolyticus*, and *Staphylococcus saprophyticus*. During testing, the method of serial dilutions proved that the extract of *Lavatera thuringiaca* L. has a bactericidal effect on *Staphylococcus aureus* at a concentration of 0.83 *μ*g/*μ*l, on *Escherichia coli* at a concentration of 3.33 *μ*g/*μ*l, on *Pseudomonas aeruginosa* at a concentration of 0.83 *μ*g/*μ*l, on *Streptococcus pneumoniae* at a concentration of 1.67 *μ*g/*μ*l, on a clinical isolate of *Staphylococcus haemolyticus* at a concentration of 26.65 *μ*g/*μ*l, on *Staphylococcus saprophyticus* at a concentration of 6.67 *μ*g/*μ*l, and against *Klebsiella pneumoniae* at a concentration of 13.36 *μ*g/*μ*l. The test result showed that the extract also has fungicidal activity against the test culture of *Candida albicans* at a concentration of 0.21 *μ*g/*μ*l. At tests, the disc diffusion method proved that the extract has antimicrobial activity with high values of the growth suppression zone exceeding 15 mm. The zones of growth retardation of the test strains were 19.33 ± 1.15 for *Staphylococcus aureus*; 17.33 ± 3.21 for *Escherichia coli*; 15.67 ± 0.57 for *Pseudomonas aeruginosa*; 20.0 ± 1.0 for *Streptococcus pneumoniae*; 16.0 ± 2.64 for *Klebsiella pneumoniae*; 15.0 ± 1.0 for *Staphylococcus saprophyticus*, and 22.0 ± 1.73 for *Candida albicans*. In relation to the clinical isolate of *Staphylococcus haemolyticus*, the extract has a bacteriostatic effect.

## 1. Introduction

In recent decades, consumption of medicinal plants and plant-based products has been steadily growing, since they are in no way inferior in quality and biological activity to synthetic drugs [1]. Herbal preparations are able to influence the human body without disrupting the course of physiological processes. By studying biologically active substances and developing medicines from plant materials of various species, it is possible to expand the range of domestic products of plant origin and meet the need for highly effective and low-toxic herbal medicines. To solve this task, it is necessary to use our own raw materials, production facilities, and scientific and technical potential. Practical interest of the species is *Lavatera thuringiaca* L., which is a promising medicinal plant rich in biologically active substances.

The genus *Lavatera* L. from the Malvaceae Juss. family is represented by 25 species found mainly in the Mediterranean countries. Only one species, *Lavatera thuringiaca* L. [2], grows almost in every part of Kazakhstan's territory. This is a promising medicinal plant used in folk medicine as an anti-inflammatory, emollient, enveloping, sedative, hemostatic, and laxative [3]. It is a herbaceous multistemmed perennial of 25–200 cm high, covered with short stellate hairs. The stems are simple or branched in the upper half. The leaves are almost rounded, cut off at the base, and 5-lobed; the upper leaves are 3-lobed; and the lobes are ovate or broadly ovate, obtuse, with a longer middle one. The flowers are solitary, large, wide open with a pink corolla and obovate, deeply bilobate petals. The seeds are reniform, dark brown, and smooth. The plant blooms in June–September. It grows in the steppe zone, in meadows, fallow lands, in thickets of bushes, on the outskirts of groves, near roads, and in low places near rivers and lakes throughout Kazakhstan. Spread area: Europe, the Mediterranean, the Balkans, the Caucasus, Central and Minor Asia, Western and Eastern Siberia, and Western China.

It is used as a technical plant for the production of fibers, suitable for the production of twine, and ropes, as well as an ornamental and honey-bearing plant [4].

Due to the high content of flavonoids, *Lavatera thuringiaca* L. is used in pharmaceutical practice [5] as an antitussive agent [6]. The following compounds were exported and identified from the flowers of *Lavatera thuringiaca* L.: kaempferol 3-О-*β*-glucoside, kaempferol, and quercetin 3-О-rutinoside, and two fractions containing different ratios of cis- and trans-tiliroside and para-coumaric acid. Using the high-performance liquid chromatography method, Russian scientists have identified the composition of the complex of phenolic compounds of vegetative organs and flowers of the *Lavatera thuringiaca* L. harvested in the Altai Territory. The roots contained derivatives of caffeic and chlorogenic acids, and umbelliferone; the grass and stems contained derivatives of chlorogenic acid, flavonoids of the flavone and catechin groups, and coumarin compounds; the leaves contained derivatives of chlorogenic and ferulic acids and derivatives of quercetin (quercitrin, etc.), kaempferol, and flavone; and the flowers contained phenologlycosides and kaempferol and flavone derivatives [7]. This plant has abundant polyphenolic compounds; flavonoids; pronounced antioxidants [8]; and anti-inflammatory [9], antimicrobial [10], antibacterial [11], cytotoxic [12], and other types of activity. The plant extracts of the genus *Lavatera* of the Malvaceae family obtained by the method of Soxhlet, maceration, microwave, ultrasonic, and subcritical water extraction are used in medicine as agents for the treatment of infectious diseases [13] and cancer [14] and also have antioxidant, cytotoxic, and antibacterial activity.

Thus, a review of the literature indicates that the research conducted by foreign scientists does not provide solid information on the presence of terpenes that determine the composition of the plant and extracts obtained from the species *Lavatera thuringiaca* L. The CO_2_-extraction method proposed for improving the extraction and selectivity of plant bioactive compounds [15] is an indispensable method for detecting terpene classes.

Earlier, foreign scientists [12] obtained extracts from the plant-based raw materials of medicinal plant *Lavatera thuringiaca* L. by methods of Soxhlet, maceration, microwave, ultrasonic, and subcritical water extraction. When determining the component composition of these obtained extracts, the class of terpenes was not identified. Flavonoids were not determined in the chemical composition of *Lavatera thuringiaca* L. extract by HPLC. The quantitative analysis carried out by the GC/MS method shows a negative result for the content of flavonoids. During this study, a thick extract of CO_2_ was obtained under subcritical conditions from the aboveground parts of *Lavatera thuringiaca* L.; along with this, the component composition was studied, 31 chemical compounds and terpenes were identified and lastly, antimicrobial activity against pathogenic bacteria was established.

## 2. Materials and Methods

### 2.1. The Plant Material

The plant material of the aboveground part of *Lavatera thuringiaca* L. was collected during its flowering phase in the Shamalgan gorge of the Zailiyskiy Alatau ridge on the territory of the Karasay district of the Almaty region in June 2018. The coordinates of the places of growth and collection of the raw material studied were determined using a GPS navigator (point 1: N 43°12′27.34″, E 76°33′55.36″, point 2: N 43°10′36.06″, E 76°31′0.45″, point 3: N 43°11′21.12″, E 76°30′37.17″).

The collected vegetable plant materials were dried at a temperature of +25 ± 5°C naturally in the shade, in a well-ventilated place. The moisture content of the plant-based raw materials does not exceed 10%. The dried plant material was crushed on a KDU–2 crusher and stored at a temperature of +15°C to 25°C and a humidity of no more than 65% in paper bags.

Plant samples were identified at the Institute of Botany and Phyto-Introduction (Almaty) (registration number of reference No. 01–08/273).

### 2.2. Preparation of Carbon Dioxide Extract

The dried above ground part of *Lavatera thuringiaca L*., which was harvested in June 2018, was used as a medicinal plant raw material for the production of carbon dioxide extraction. The extract was obtained in the production base of the limited liability partnership “production of medicines Zhanafarm.” The extract was received under subcritical conditions on a 5-L laboratory machine for CO_2_ flow-through extraction in accordance with the Company's SS 8050–85 Standard. Liquid carbon dioxide was used as an extractant. Optimal conditions for obtaining the CO_2_ extract were maintained: pressure of 51–65 atmospheres, temperature of 18–27°C, and extraction time of 7–11 hours.

To increase the specific contact surface of the extracted raw material with liquid CO_2_, the grass was crushed on the KDU-2 crusher. The particle size of the plant-based raw material is 1–3 mm. To obtain a thick carbon dioxide extract of the *Lavatera thuringiaca* L. herb, 2000 g of raw material was taken, from which 25 g of extract was obtained; that is, the yield constituted 1.25%.

#### 2.2.1. Extraction Procedure

The shredded plant material was loaded into an UUPE-5L extractor (a laboratory carbon dioxide flow extraction machine). The extraction was carried out with carbon dioxide under the conditions specified for this process. Liquefied carbon dioxide was delivered from the storage tank using a high-pressure pump at a pressure of 6 MPa and entered the extractor tank. From the extractor, the flow with the substances dissolved in carbon dioxide was fed to the collectors, where the process of separating the solvent and solute was carried out with sequential pressure relief. The extract was deposited into collectors and then discharged as a finished product.

### 2.3. Determination of the Component Composition of the Extract

Gas chromatography with mass spectrometric detection (Agilent 7890A/5975C) was used for the qualitative and quantitative analysis of the carbon dioxide extract of *Lavatera thuringiaca* L. Chromatographic analysis conditions are as follows: sample volume of −1.0 *μ*l, sample injection temperature of −240°С, and flow division of –1 : 10. Separation was carried out using a 30-metre long WAXetr chromatographic capillary column, with an inner diameter of 0.25 mm and a film thickness of 0.25 *μ*m at a constant gas carrier (helium) velocity of 1 ml/min. The chromatographic temperature was programmed from 40°C (0 min exposure) to 260°C with a heating rate of 10°C/min (20 min exposure). Detection was performed in the SCAN *m*/*z* 34–850 mode. Agilent MSD ChemStation software (version 1701EA) was used to control the gas chromatography system, register the results, and process the data. Data processing included the determination of retention times, peak areas, and verification of spectral information obtained with the mass spectrometric detector.

#### 2.3.1. Identification of the Component Composition of the Extract

To identify the obtained mass spectra, we used the Wiley 7^th^ edition and NIST′02 data libraries. The components were identified by the mass spectrum and retention time.

#### 2.3.2. Quantitative Determination

For the quantitative determination of terpenes in the CO_2_ extract, the gas chromatography method with mass spectrometric detection (Agilent 7890A/5975C) was used. The percentage of components was calculated automatically based on the peak areas of the total ion chromatogram.

The content of the sum of terpenes in the extract should be at least 50.0%.

### 2.4. Determination of Antimicrobial Activity

To determine the antimicrobial activity, standard test strains of microorganisms were used: *Pseudomonas aeruginosa* ATCC 9027, *Candida albicans* ATCC 10231, *Escherichia coli* ATCC 8739, *Streptococcus pneumonia* ATCC 660, and *Klebsiella pneumoniae* ATCC 700603, obtained from the American Type Culture Collection (ATCC, USA), and *Staphylococcus aureus* ATCC 6538-P, *Staphylococcus haemolyticus*, and *Staphylococcus saprophyticus*, obtained from the Republican Collection of Microorganisms (Nur-Sultan, Kazakhstan).

Studies of the sensitivity of microorganisms were carried out on standard nutrient media:

- Mueller–Hinton medium: Mueller–Hinton agar (M173), HiMedia, India
- Mueller–Hinton broth (M391), HiMedia, India
- Sabouraud liquid medium (M033), HiMedia, India

The antimicrobial activity of the carbon dioxide extract of *Lavatera thuringiaca* L. was determined by two methods: the method of serial dilution and the disk-diffusion method.

#### 2.4.1. Definition of the Extract's Antimicrobial Activity by the Serial Dilution Method

To determine antimicrobial activity, a 96-well plate was used. In all wells, except for the first ones, it was required to add the Mueller–Hinton nutrient broth (M391) (for testing bacteria) and Sabouraud broth (for testing fungi), in the amount of 100 *μ*l (from the 1^st^ to the 12^th^ wells). The extract was preliminarily dissolved in 0.5 ml of 0.9% sodium chloride solution and introduced in the volume of 100 *μ*l into the 1^st^ well, making serial dilutions by taking the mixture (Mueller–Hinton broth/Sabouraud-dextrose broth (100 *μ*l) + test drug (100 *μ*l)) from the 1^st^ well in the amount of 100 mcl into the 2^nd^ well, already containing 100 mcl of the broth. The test sample was thoroughly mixed and then 100 *μ*l of it was transferred into the broth from the 2^nd^ well to the 3^rd^ well, which also initially contained 100 *μ*l of the broth. This procedure was repeated until the required number of dilutions was reached. 50 *μ*l of the mixture was removed from the last well. Thus, the following dilutions were obtained: 1 : 1, 1 : 2; 1 : 4, 1 : 8, 1 : 16, 1 : 32, 1 : 64, 1 : 128, 1 : 256, 1 : 512, 1 : 1024, which corresponds to the wells from 1 to 11. The 12^th^ well was for the test strain control.

**Figure 1:**
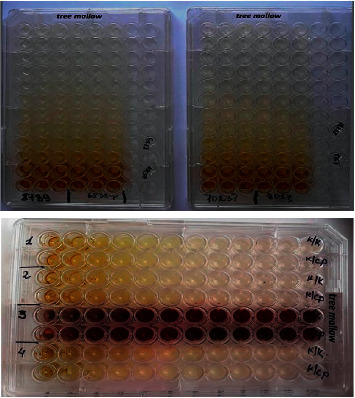
Setting antimicrobial activity in a 96-well plate.

After a series of dilutions, 20 *μ*l of test strains of microorganisms at a concentration of 1.5 × 10^6^ CFU/ml was added to all wells (Figure 1).

All the samples were incubated for 18–24 hours at 37 ± 1°C. After the incubation time, the samples were plated on Petri dishes with Mueller–Hinton nutrient medium to determine living cells. The results were taken into account by the presence of visible growth of microorganisms on the surface of a dense nutrient medium.

The minimum bactericidal concentration (MBC) was considered the lowest concentration in a test tube that suppressed the growth of microorganisms.  Table 1 shows the labelling of Petri dishes according to dilutions.

#### 2.4.2. Determination of Antimicrobial Activity by the Disk-Diffusion Method

The disk-diffusion method was carried out by applying the discs treated with the preparation on Petri dishes using sterile forceps at a distance of 15–20 mm from the dish edge and from each other. The Petri dishes were preinoculated with the test strains suspension with a density of 1.5 × 10^8^ CFU/ml. For inoculation, sterile cotton swabs were immersed in the suspension and then slightly pressed against the test tube walls and streaked in three directions, turning the cup by 60°. Сartridges with ready-made sterile discs (HiMedia) were used in the study. The disks were preliminarily saturated with the extract within the exposure time of approximately 30 min.

After inoculation, the dishes were placed in a thermostat for incubation of bacteria for 18–24 hours at 37°C. The results of the disc diffusion method were taken into account by calculating zones diameter of retardation/suppression growth with an accuracy of 1 mm (Performance Standards for Antimicrobial Susceptibility Testing, 2015; Guidelines for determining the sensitivity of microorganisms to antibacterial drugs, 2004; Reference Method for Broth Dilution Antifungal Susceptibility Testing of Yeast, 2017) [16–18].

## 3. Results and Discussion

### 3.1. Determination of the Component Composition of the CO_2_ Extract

To obtain the extract, we analyzed the extraction capacity of carbon dioxide under sub- and supercritical conditions and various physical parameters. The results are shown in Table 2.

The study shows that out of all 5 obtained carbon dioxide extracts of *Lavatera thuringiaca* L. under subcritical conditions, according to the output data parameters, chemical composition, and technological parameters, extract No.1 is optimal. Thus, when obtaining an extract from the herb *Lavatera thuringiaca* L. it was found that the yield of the extract under subcritical conditions is significantly higher than other production methods (supercritical CO_2_ extraction). For comparison, we took one sample of the extract obtained under supercritical conditions.

**Figure 2:**
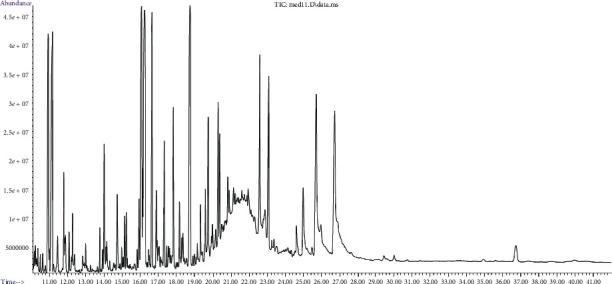
The analysis chromatogram of the *Lavatera thuringiaca* L subcritical СО_2_ extract.

Figure 2 and Table 3 show the results of a study of the chemical composition of a thick extract obtained from the medicinal plant raw material *Lavatera thuringiaca* L. The content of 31 compounds was determined by chromatography-mass spectrometry. We studied the pharmacological activity of these compounds by analyzing the works of foreign scientists.

Monoterpenes: isopulegone 0.7%, pulegone 5.08%, and verbenone 1.93%; diterpenes: phytol 2.49%; sesquiterpenes: bisabolol oxide A 8.26%, *α*-bisabolol oxide B 9.65%, *γ*-muurolene 0.75%, spathulenol 6.97%, cis-*β*-farnesene 7.63%, and alloaromadendrene 0.4%; fatty acids: linolenic acid 9.38%, linoleic acid 6.95%, elaidic acid 2.57%, stearic acid 1.14%, myristic acid 2.33%, palmitic acid, and ethyl ester 2.72%; and coumarins: herniarin or 7-methoxycoumarin 5.61% were found among the main classes of compounds CO_2_ extract of *Lavatera thuringiaca* L.

### 3.2. Results of Determination of the Antimicrobial Activity of the CO_2_ Extract

Antimicrobial activity was studied on the CO_2_ extract of *Lavatera thuringiaca* L. The main active ingredients of the extract *Lavatera thuringiaca* L. are as follows: compounds of terpenes 53.3%, fatty acids 28.55%, and coumarins 5.61% with a supposed antimicrobial effect.

When studying the antimicrobial activity by the method of serial dilutions, the antibacterial and fungicidal effect of CO_2_ extract of *Lavatera thuringiaca* L. was established in relation to the analyzed strains of microorganisms *Staphylococcus aureus* ATCC 6538-P, *Escherichia coli* ATCC 8739, *Pseudomonas aeruginosa* ATCC 9027, *Candida albicans* ATCC 10231, *Streptococcus pneumoniae* ATCC 660, *Klebsiella pneumoniae* ATCC 700603, *Staphylococcus haemolyticus*, and *Staphylococcus saprophyticus* (Table 4).

The results of the study of antimicrobial activity by serial dilution showed that the CO_2_ extract of *Lavatera thuringiaca* L. has the greatest bactericidal and bacteriostatic effectiveness against *Candida albicans* at a concentration of 0.21 mcg/ml; *Pseudomonas aeruginosa* at a concentration of 0.83 *μ*g/*μ*l; *Staphylococcus aureus* at a concentration of 0.83 *μ*g/*μ*l; *Streptococcus pneumoniae* at a concentration of 1.67 *μ*g/*μ*l; *Escherichia coli* at a concentration of 3.33 *μ*g/*μ*l; *Staphylococcus saprophyticus* at concentrations of 6.67 *μ*g/*μ*l and 3.33 *μ*g/*μ*l; *Klebsiella pneumoniae* at concentrations of 13.36 *μ*g/*μ*l and 6.67 *μ*g/*μ*l; and *Staphylococcus haemolyticus* at concentrations of 26.65 *μ*g/*μ*l and 13.36 *μ*g/*μ*l.

Studying the antimicrobial activity of the CO_2_ extract of *Lavatera thuringiaca* L. was carried out by the method of serial dilutions and the antimicrobial activity of the extract was established by the disk-diffusion method (Table 5).

The testing by the disk-diffusion method revealed that the extract had an antimicrobial activity with high values of the growth suppression zone exceeding 15 mm. The growth inhibition zones of the test strains were 19.33 ± 1.15 against S*taphylococcus aureus* ATCC 6538-p., 17.33 ± 3.21 against *Escherichia coli* ATCC 8739, 15.67 ± 0.57 against *Pseudomonas aeruginosa* ATCC 9027, 20.0 ± 1.0 against *Streptococcus pneumoniae* ATCC 660, 16.0 ± 2.64 against *Klebsiella pneumoniae* ATCC 700603, and 15.0 ± 1.0 against *Staphylococcus saprophyticus*. Also, this extract has a bacteriostatic effect against the clinical isolate of *Staphylococcus haemolyticus* and fungicidal activity against *Candida albicans* ATCC 10231 with a growth retardation zone of 22.0 ± 1.73. When interpreting the data, it was conditionally assumed that the diameter of the growth retardation zone of more than 15 mm proves high antimicrobial activity; 10–15 mm, average antimicrobial activity; and less than 10 mm, low antimicrobial activity [60].

The antimicrobial activity of the obtained extracts was compared with other extracts obtained by Serbian scientists Pavle Z. Mašković and others. Five different extractive methods were used to obtain the extracts: Soxhlet, maceration, ultrasonic, microwave, and subcritical water extraction. The component composition of these extracts was determined by the HPLC-DAD method. The antibacterial activity of these extracts was tested in vitro against the following Gram-positive bacteria: *Staphylococcus saprophyticus, Staphylococcus aureus*, *Listeria ivanovii*, *Listeria inocun*, *Enterococcus faecalis*, *Listeria monocytogenes*, *Bacillus spizizenii*, and *Enterococcus faecium*, as well as the following Gram-negative bacteria: *Escherichia coli*, *Salmonella enteritidis*, *Enterobacter aerogenes*, *Citrobacter freundii*, *Salmonella typhimurium*, *Pseudomonas aeruginosa*, and *Proteus mirabilis*. According to the results of these studies, the highest activity of the extract obtained by subcritical water extraction was observed for *Staphylococcus saprophyticus* (at a concentration of 7.81 *μ*g/*μ*l). Ultrasonic extraction had the strongest effect on *Salmonella typhimurium* (in the concentric 7.81 *μ*g/*μ*l). Maceration extraction had a strong effect on *Enterobacter aerogenes* (at a concentration of 15.82 *μ*g/*μ*l), *Proteus mirabilis* (at a concentration of 7.81 *μ*g/*μ*l, and *Staphylococcus saprophyticus* (at a concentration of 15.82 *μ*g/*μ*l). Soxhlet extract has been proven to be highly effective against *Salmonella enteritidis*.

A comparative analysis of our data with the data obtained by Serbian scientists shows the following results: the extract obtained by Serbian scientists by ultrasonic extraction shows a high bactericidal ability on *Staphylococcus aureus* at a concentration of 31.25 *μ*g/*μ*l; the extract obtained by Serbian scientists using microwave and subcritical water extraction methods shows bactericidal activity on *Staphylococcus aureus* at a concentration of 62.50 *μ*g/*μ*l, and in the extract obtained by CO_2_ extraction under subcritical conditions, the bactericidal activity on *Staphylococcus aureus* was proven at a concentration of 0.83 *μ*g/*μ*l; the extract obtained by Serbian scientists by ultrasonic extraction acts on *Pseudomonas aeruginosa* at a concentration of 15.82 *μ*g/*μ*l; the extract obtained by Serbian scientists using microwave and subcritical water extraction methods acts on *Pseudomonas aeruginosa* at a concentration of 62.50 *μ*g/*μ*l; in the extract obtained by CO_2_ extraction under subcritical conditions, the bactericidal activity on *Pseudomonas aeruginosa* is at a concentration of 0.83 *μ*g/*μ*l; the extract obtained by Serbian scientists using ultrasonic extraction and maceration methods acts on *Escherichia coli* at a concentration of 62.50 *μ*g/*μ*l; and in the extract obtained by CO_2_ extraction under subcritical conditions, the bactericidal activity on *Escherichia coli* is at a concentration of 3.33 *μ*g/*μ*l. According to the presented data, the extract obtained by CO_2_ extraction under subcritical conditions has a higher antimicrobial activity than the extract obtained by Serbian scientists using the following methods: ultrasonic, subcritical water, microwave, and maceration extraction (Table 6).

## 4. Conclusion

The optimal conditions for CO_2_ extraction of carbon dioxide extract from *Lavatera thuringiaca* L. medicinal plant raw materials have been developed. The yield of the resulting finished product is 25 g (1.25%). This method improves the quality of the finished product. The study of subcritical CO_2_ extracts has shown that they do not contain ballast substances and organic solvent residues. They contain natural biologically active substances that are not exposed to temperature and chemical influences, as well as having antimicrobial activity.

To study the phytochemical composition of the *Lavatera thuringiaca* L. medicinal plant material, thick extracts were obtained by the CO_2_-extraction method and the chemical composition was determined by gas chromatography with the mass spectrometric detector. As a result, a large amount of terpenes, coumarins, and polyunsaturated fatty acids were detected in the subcritical CO_2_ extract. 31 components of the group of monoterpenes, diterpenes, sesquiterpenes, fatty acids, and coumarins from raw materials of *Lavatera thuringiaca* L. were identified.

The antibacterial activity of the CO_2_ extract of *Lavatera thuringiaca* L. obtained under subcritical conditions was determined. The tested sample of the *Lavatera thuringiaca* L. extract exhibits antimicrobial activity against *Staphylococcus aureus* ATCC 6538-р, *Escherichia coli* ATCC 8739, *Pseudomonas aeruginosa* ATCC 9027, *Streptococcus pneumoniae* ATCC 660, *Staphylococcus haemolyticus*, *Staphylococcus saprophyticus*, and *Klebsiella pneumoniae* ATCC 700603 both by serial dilution in broth and by diffusion testing in agar, forming the zones of test strains growth inhibition. This sample also exhibits fungicidal activity against the *Candida albicans* ATCC 10231 test culture by serial dilution and agar diffusion.

The *Lavatera thuringiaca* L. extract was active against all the test strains (both museum and clinical). It has been experimentally shown that the extract of *Lavatera thuringiaca* L., when tested by the serial dilutions method, has a bactericidal effect at the following dilutions: on *Staphylococcus aureus* at a concentration of 0.83 *μ*g/*μ*l, on *Escherichia coli* at a concentration of 3.33 *μ*g/*μ*l, on *Pseudomonas aeruginosa* at a concentration of 0.83 *μ*g/*μ*l, on *Streptococcus pneumoniae* at a concentration of 1.67 *μ*g/*μ*l, on a clinical isolate of *Staphylococcus haemolyticus* at a concentration of 26.65 *μ*g/*μ*l, for *Staphylococcus saprophyticus* at a concentration of 6.67 *μ*g/*μ*l, and for *Klebsiella pneumoniae* at a concentration of 13.36 *μ*g/*μ*l. The data obtained from testing for the determination of fungicidal activity showed that the extract has activity against the test culture of *Candida albicans* at a concentration of 0.21 *μ*g/*μ*l. In addition, when tested by the disk-diffusion method, it was also found that the extract had an antimicrobial activity with high values of the growth suppression zone exceeding 15 mm. The growth inhibition zones of the test strains were 19.33 ± 1.15 against S*taphylococcus aureu*s, 17.33 ± 3.21 against *Escherichia coli*, 15.67 ± 0.57 against *Pseudomonas aeruginosa*, 20.0 ± 1.0 against *Streptococcus pneumoniae*, 16.0 ± 2.64 against *Klebsiella pneumoniae*, 15.0 ± 1.0 against *Staphylococcus saprophyticus*, and 22.0 ± 1.73 against *Candida albicans*. Also, this extract has a bacteriostatic effect against the clinical isolate of *Staphylococcus haemolyticus*.

The results obtained for the test sample indicate the prospects for further study of the *Malva thuringiaca* (*Lavatera thuringiaca* L.) for medical practice.

## Figures and Tables

**Table 1:** Labelling of Petri dishes according to dilutions.

No. of wells	Well 1	Well 2	Well 3	Well 4	Well 5	Well 6	Well 7	Well 8	Well 9	Well 10	Well 11	Well 12
Dilution of the test sample and Mueller–Hinton broth/Sabouraud broth	1 : 1	1 : 2	1 : 4	1 : 8	1 : 16	1 : 32	1 : 64	1 : 128	1 : 256	1 : 512	1 : 1024	Test strain control

**Table 2:** Extract yield at different parameters of subcritical and supercritical extraction.

Extraction samples obtained (thick extract)	Parameters				Extract yield (g) (%)
	Weight of the plant material (g)	Working pressure (atmospheres)	Extraction process temperature (°С)	Extraction progress time (hours)	
Subcritical CO_2_ extraction (thick extract)					
No. 1	2,000	51	21	11	25 (1.25)
No. 2	1,900	56	18	7	12 (0.63)
No. 3	1,850	60	20	9	10 (0.54)
No. 4	1,800	63	21	10	7 (0.38)
No. 5	1,700	65	21	11	6 (0.35)
Supercritical CO_2_ extraction (thick extract)					
No. 1	2,000	98.69	50	More than 1	12 (0.63)

**Table 3:** The results of the chromatographic analysis of the *Lavatera thuringiaca* L. subcritical CO_2_ extract.

No.	RT (min)	Name of compound	The class	Activity	PubChem CID	(%)
1	10.2	Isopulegone	Monoterpenoid	Anticonvulsant, anti-inflammatory, antioxidant, gastroprotective, antistress [19–21]	34645	0.7
2	10.2	D-Menthol	Terpene	Anti-irritant, pain-reliever [22–24]	165675	0.8
3	10.5	Alloaromadendrene	Sesquiterpene	Antioxidant [25]	42608158	0.4
4	10.9	Pulegone	Monoterpenе	Antinociceptive [26]	442495	5.08
5	11.2	Cis-*β*-farnesene	Sesquiterpene	Antifungal [27]	5317319	7.63
6	11.2	Humulene	Terpene	Anti-inflammatory, appetite suppressant, pain reliever [28, 29]	5281520	0.19
7	14.0	Verbenone	Monoterpenе	Antimicrobial, antifungal, anticonvulsive [30–32]	29025	1.93
8	14.7	Caryophyllene oxide	Terpene	Anticancer and analgesic, anti-inflammatory [33, 34]	1742210	1.35
9	15.2	E-Nerolidol	Terpene	Antifungal, sedative [35, 36]	5281525	0.80
10	16.1	Spathulenol	Sesquiterpenoid	Antioxidant, anti-inflammatory, antiproliferative, and antimycobacterial [37]	92231	6.97
11	16.2	*α*-Bisabolol oxide B	Sesquiterpene	Anti-irritant, anti-inflammatory, and antimicrobial [38–41]	6432283	9.65
12	16.9	*α*-Bisabolol	Terpene	Anti-irritant, anti-inflammatory, antimicrobial, analgesic [38–41]	10586	1.36
13	18.7	Bisabolol oxide A	Sesquiterpene	Anti-irritant, anti-inflammatory, and antimicrobial [38–41]	13092559	8.26
14	17.3	Palmitic acid, ethyl ester	Fatty acid	Anti-inflammatory [42]	12366	2.72
15	20.3	Linolenic acid, ethyl ester	Fatty acid	Promitogeinc and activating effects on hepatic stellate cells (HSC), anticancer [43, 44]	5367460	3.15
16	20.4	Phytol	Terpene	Anti-inflammatory, analgesic [45, 46]	5280435	2.49
17	20.9	Myristic acid	Saturated fatty acid	Antimicrobial [47]	11005	2.33
18	23.1	Herniarin or 7-methoxycoumarin	Coumarin	Antigenotoxic, anti-inflammatory, antinociceptive [48–50]	10748	5.61
19	24.6	Stearic acid	Saturated fatty acid	Anticancer [51]	5281	1.14
20	25.0	Elaidic acid	Saturated fatty acid	Against herpesviruses [52]	637517	2.57
21	25.7	Linoleic acid	Polyunsaturated fatty acid	Anti-inflammatory, antibacterial [53–55]	5280450	6.95
22	26.7	Linolenic acid	Fatty acid	Inflammatory, antioxidant, cytotoxic, antibacterial, and antifungal [53, 54, 56]	5280934	9.38
23	36.7	Cannabidiol	Phytocannabinoid	Anticonvulsant [57]	644019	0.96
24	11.5	*γ*-Muurolene	Sesquiterpene	Antimicrobial, anti-inflammatory [58]	12313020	0.75
25	11.9	Piperitone	Monoterpenoid	Antimicrobial, antiviral, perfume compositions [59]	6987	0.98
26	16.1	Perhydrofarnesyl acetone	Diterpenoids	Perfume composition	10408	1.36
27	10.4	Isocaryophyllene	Sesquiterpene	Antifungal	5281522	0.36
28	11.0	Isovaleric acid	Fatty acid	Sedative, used in the production of validol, valocordin	10430	0.31
29	12.4	*α*-Curcumene	Sesquiterpene	Antimicrobial, anti-inflammatory, antifungal	92139	0.35
30	14.1	Geranyl linalоol	Diterpene	Antimicrobial, anti-inflammatory	5365872	0.39
31	16.5	Thymol	Monoterpenе	Antiseptic, antibacterial, and antifungal	6989	0.54

**Table 4:** The antimicrobial activity results of the extract (CO_2_ extraction) obtained by the method of serial dilution.

Test strains	Minimum dilution of the thuringian tree mallow extract (*μ*g/*μ*l)	
	Bactericidal action	Bacteriostatic action
*Staphylococcus aureus* ATCC 6538-Р	0.83	0.83
*Escherichia coli* ATCC 8739	3.33	3.33
*Pseudomonas aeruginosa* ATCC 9027	0.83	0.83
*Candida albicans* ATCC 10231	0.21	0.21
*Streptococcus pneumoniae* ATCC 660	1.67	1.67
*Klebsiella pneumoniae* ATCC 700603	13.36	6.67
*Staphylococcus haemolyticus*	26.65	13.36
*Staphylococcus saprophyticus*	6.67	3.33

**Table 5:** The antimicrobial activity results of the extract (CO_2_ extraction) obtained by the disc diffusion method.

Test sample	Minimum bactericidal concentration (1 : 1 extract dilution)
*Staphylococcus aureus* ATCC 6538-Р	19.33 ± 1.15
*Escherichia coli* ATCC 8739	17.33 ± 3.21
*Pseudomonas aeruginosa* ATCC 9027	15.67 ± 0.57
*Candida albicans* ATCC 10231	22.0 ± 1.73
*Streptococcus pneumoniae* ATCC 660	20.0 ± 1.0
*Klebsiella pneumoniae* ATCC 700603	16.0 ± 2.64
*Staphylococcus haemolyticus*	—
*Staphylococcus saprophyticus*	15.0 ± 1.0

**Table 6:** Comparative analysis of the results of antimicrobial activity of extracts obtained by extraction methods.

Test sample	Minimum concentration of *Lavatera thuringiaca* L. extracts (*μ*g/*μ*l)				
	Ultrasonic extraction	Subcritical water extraction	Microwave extraction	Maceration extraction	CO_2_ extraction under subcritical conditions
*Staphylococcus aureus*	31.25	62.50	62.50	125	0.83
*Pseudomonas aeruginosa*	15.82	62.50	62.50	250	0.83
*Escherichia coli*	62.50	125	250	62.50	3.33

## Data Availability

The data used to support the findings of this study are available upon request.
